# Characteristics, course and outcome of patients receiving physiotherapy in primary health care in Norway: design of a longitudinal observational project

**DOI:** 10.1186/s12913-018-3729-y

**Published:** 2018-12-04

**Authors:** Kari Anne I. Evensen, Hilde Stendal Robinson, Ingebrigt Meisingset, Astrid Woodhouse, Marit Thielemann, Wenche S. Bjorbækmo, Gard Myhre, Anne E. Hansen, Ottar Vasseljen, Nina K. Vøllestad

**Affiliations:** 1 Department of Public Health and Nursing, Norwegian University of Science and Technology (NTNU), Trondheim, Norway; 2 Department of Clinical and Molecular Medicine, NTNU, Trondheim, Norway; 3 Department of Health Sciences, University of Oslo, Oslo, Norway; 4 Unit for Physiotherapy Services, Trondheim Municipality, Trondheim, Norway; 5 Department of Circulation and Medical Imaging, NTNU, Trondheim, Norway; 6 Norwegian Advisory Unit on Complex Symptom Disorders, St. Olavs hospital, Trondheim University Hospital, Trondheim, Norway; 7 Department of Physiotherapy, Oslo Metropolitan University, Oslo, Norway

**Keywords:** Physiotherapy, Primary health care services, Electronic registration, Database, Cohort, FYSIOPRIM, Municipality

## Abstract

**Background:**

Physiotherapists (PTs) in primary health care manage patients with large variation in medical diagnosis, age, functional status, disability and prognosis. Lack of knowledge and systematically collected data from patients treated by PTs in primary health care has prompted this longitudinal observational physiotherapy project. This paper aims to describe a method for developing a database of patients managed by PTs in primary health care, with the main purpose to study patients’ characteristics, treatment courses and prognostic factors for favourable outcome.

**Methods:**

This is a longitudinal observational project, following patients through their physiotherapy treatment periods in primary health care in Norway and until one year after inclusion. The project involves both private practitioners and municipally employed PTs working in primary health care in nine municipalities in Norway. The patients are recruited to three different cohorts depending on age and whether they are referred to a private practitioner or a municipally employed PT. All data are recorded electronically, transferred and stored securely. For all patients we have included extensive questionnaires to obtain information about demographics, disability and function, pain-related variables, psychosocial factors, treatments and evaluation of treatment as well as response to clinical tests. The PTs have access to use their own patients’ data. We have also prepared for linkage to national patient registers and data collected in population-based studies to be able to gather further important data.

**Discussion:**

This project will have important implications for physiotherapy services in primary health care. The database contains more than 3000 patients, and data collection is ongoing. Data collected so far suggest that the patients included are representative of the larger population of patients treated by private practitioners or municipally employed PTs in Norway. This large scale prospective physiotherapy project will provide knowledge about the patient groups, applied treatments and short- and long-term outcome of the patients.

**Trial registration:**

ClinicalTrials.gov Identifier: NCT03626389. Registered on August 13th 2018 (retrospectively registered).

**Electronic supplementary material:**

The online version of this article (10.1186/s12913-018-3729-y) contains supplementary material, which is available to authorized users.

## Background

There is a lack of comprehensive and systematically collected data about patients receiving primary care physiotherapy. Physiotherapists (PTs) in primary health care manage patients with large variation in medical diagnosis, age, functional status, disability and prognosis [1]. Among PTs in private practice in Norway, information about patients and treatments is limited to diagnoses, number of treatments and costs, which the PTs are obliged to report to the Norwegian Directorate of Health in order to receive reimbursement. Apart from this, little is known about the complaints for which patients seek or receive physiotherapy services. Comparative knowledge of the different patient groups, applied treatments, clinical courses and outcome measures is missing. Hence, there is a need for robust and comprehensive data of how and to whom the primary care physiotherapy services are delivered, and whether the treatment goals are achieved. This includes systematically collected information about prognostic factors, content and effect of commonly applied treatments. This knowledge will aid health care managerial decision making and policy makers in prioritising among health care services, and to improve rehabilitation of patients in primary health care.

In Norway, one large and some smaller local studies of patients treated by PTs in primary health care have been published [2–4]. All are cross-sectional studies and only a limited number of factors are studied. In the largest study, the PTs answered questions about sex, age, diagnosis, treatment modalities and main goals for treatment for a total of 3196 patients [4]. Patient-reported outcome measures (PROMs) were not used, and none of the studies were designed to describe clinical courses or prognostic factors. An international study used registry data from three different countries (USA, Netherlands and Israel) [1]. Sex, age, diagnoses, affected body regions, duration of complaints as well as number and type of treatment (modality) were registered. The study did, however, not include PROMs as all data were recorded by the PTs. In a study of 7670 patients with musculoskeletal complaints seeking physiotherapy treatment in New Zealand, sex, age, affected body region, patient-reported pain intensity and disability as well as number of treatments were recorded [5]. The study showed clear reduction in pain intensity and disability after treatment. No other outcome measures were recorded, nor was there any information on effects and course of symptoms after treatment. Follow-up data after treatment and a wider spectrum of baseline prognostic factors and outcome measures are needed for comparative exploration of symptom course and treatment outcome in the different patient groups receiving primary care physiotherapy.

Through the Research Program for Physiotherapy in Primary Health Care, the FYSIOPRIM, we have put together a set of standardised methods and tools to enable systematic studies of clinical courses for patients treated by PTs in primary health care in Norway. This paper describes the framework, design and methods used for the systematic data collection, where the purpose is to establish a database for studying patient characteristics, prognostic factors, applied treatments and outcome in various patient groups. The patients include children and adults of all ages receiving primary care physiotherapy services from private practitioners and municipally employed PTs. This large scale prospective physiotherapy project will provide knowledge about the patient groups, applied treatments, and short- and long-term outcome of the patients.

## Methods/design

### Aims

The primary aim of the project is to build a database by systematically collecting data from baseline throughout the treatment period and beyond, including PROMs, the patients’ and PTs’ main goals and plans for treatment, assessment of goal achievement and outcome. This will enable detailed descriptions of patients receiving physiotherapy services, goal-setting, type of treatments, and how general health, physical function and relevant clinical factors change throughout and after the treatment period. Secondly, we want to study associations and interaction effects between clinical characteristics, treatments and outcome, along with health-economic evaluations. We will also examine how physiotherapy practice is affected by being exposed to systematic registration of clinical data. Moreover, we will be able to compare patients receiving physiotherapy services with information from national patient registries. In addition, physical fitness, balance and walking ability will be examined in a group of healthy older adults to study associations between these aspects of physical functioning and health-related quality of life, self-reported physical and psychological functioning as well as response to physical fitness tests. We will compare data from this group with data from patients with musculoskeletal complaints who receive physiotherapy treatment, and with similar data from large population-based studies in Norway. Finally, this project enables methodological studies to validate clinical tests and questionnaires.

### Design and setting

This is a longitudinal observational project following patients through physiotherapy treatment periods in primary health care in Norway. Data are collected prospectively from the first consultation and until 1 year after baseline. The project started its data collection in June 2015, and will continue through June 2020. The project involves both private practitioners and municipally employed PTs working in primary health care in nine municipalities of Norway; Oslo, Drammen, Ski, Kongsberg, Stavanger, Trondheim, Orkanger, Rindal, and Alta. All five health regions of Norway are represented.

The Norwegian health care system is publicly funded. Most PTs working as private practitioners have a legal agreement to practice within the municipality. They are partly paid by the municipality (as a fixed financial support for practice), partly by a fee for service reimbursement by the Norwegian Health Economics Administration (HELFO), and the patient’s deductible fee (maximum around 2000 NOK per patient per calendar year). Patients seeking physiotherapy services in private practice will normally meet at the PT’s clinic.

Municipally employed PTs are on fixed salary with no costs to the patients. They work in an out-patient setting, and the patients receive physiotherapy services in their usual daily environment, i.e. for children at home, in kindergarten or at school, and for older patients often in their own home. There are no clear guidelines for determining which patients should receive physiotherapy from a private practitioner or a municipally employed PT. The choice can be based on previous personal experiences with physiotherapy, the possibility to visit a clinic, evaluation of needs and/or benefits of treating the patients in their own setting.

Patients in Trondheim are recruited to three different cohorts depending on age and whether they are referred to a private practitioner or to a municipally employed PT. In the rest of the country, patients are recruited from private practitioners only.

### Description of the cohorts

The three cohorts in the project are:

*Cohort 1: Adult patients seeking physiotherapy services in private practice*

This part of the project started its data collection in June 2015, and data collection is planned to continue through June 2020. From June 2015 through December 2017, data were collected from a total of 2754 patients above the age of 18 years seeking physiotherapy treatment from 111 PTs in nine municipalities in different parts of Norway, including 536 patients already in treatment. Among the 111 PTs, 78 (70%) were general PTs, 20 (18%) were manual therapists and 13 (12%) were PTs with special education in psychomotor physiotherapy. Seventy-seven (69%) of the PTs were working in Trondheim and recruited 2302 (84%) of the patients.

*Cohort 2: Adult and older patients receiving physiotherapy services in Trondheim Municipality*

This part of the project collected data from the beginning of May 2016 to the end of May 2018. From January 2016 till the end of September 2017, data were collected from 655 adult and older patients receiving physiotherapy services from approximately 55 PTs working in the municipality of Trondheim. The patients may be referred from general practitioners, occupational therapists, health professionals at health care centers, rehabilitation centers or hospitals, or by proxy. The physiotherapy services include “early intervention”, “rehabilitation of activities of daily living (ADL rehabilitation)” or “reablement”, and “regular physiotherapy”, all of which are typically delivered in a home setting. Early intervention is a collaboration between health and welfare centers and units for physiotherapy and occupational services. It is offered to patients in need of limited services who are referred to municipality health care services for the first time. ADL rehabilitation/reablement is offered to patients receiving home based services and who are at risk of functional deterioration, while regular physiotherapy is offered to patients in need of more specific physiotherapy services.

*Cohort 3: Children receiving physiotherapy services in Trondheim Municipality*

This part of the project collected data from May 2016 through April 2017. During this period, data were collected from 162 children aged 0–18 years receiving physiotherapy services from approximately 25 PTs working in the municipality of Trondheim. For children, physiotherapy may be initiated on referral from parents, personnel in kindergarten or school, general practitioners, occupational therapists, and health professionals at primary health care centers or hospitals. The children typically receive physiotherapy services at primary health care centers, in their own home, in kindergarten, or at school, depending on the child’s usual primary location and condition.

### Data collection

**Fig. 1:**
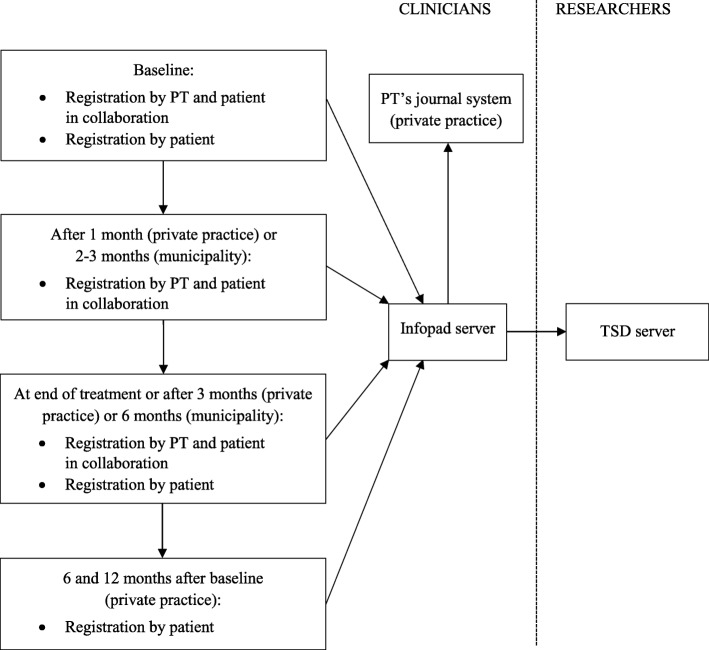
Flowchart of data collection and data users in FYSIOPRIM *FYSIOPRIM* Research Program for Physiotherapy in Primary Health Care, *TSD* Services for Sensitive Data, *PT* Physiotherapist

A flowchart of the data collection is presented in Fig. 1. Patients are asked to participate on their first encounter with a PT in primary health care. Project information and consent forms are available in Norwegian and English. We collect baseline data in two steps. First, data are registered by the PT and the patient in collaboration. They jointly agree on the main treatment goal and plan for treatment. The PT asks the patient to define and score their most important specific functional problems using the Patient-Specific Functional Scale (PSFS) [6]. The PT registers the patients’ referral and diagnosis, and determines whether the patient should fill in any disease- or region-specific questionnaires. Secondly, the patient completes questionnaires either by using an e-tablet or through a web-link sent by e-mail.

Approximately 1 month after baseline (for patients seeking physiotherapy services from private practitioners), or 2–3 months after baseline (for patients receiving physiotherapy services from municipally employed PTs), the PT and the patient together evaluate goal achievement and fulfillment of the treatment plan so far. They adjust goals and plans if needed, and the patient recompletes the PSFS. At end of treatment, or maximum 3 months (in private practice) or 6 months after baseline (in the municipality), the PT and the patient again evaluate goal achievement and fulfillment of the treatment plan. The patient then completes the same standardised questionnaires as at baseline. The same questionnaires are completed 6 and 12 months after baseline by patients in private practice using a web link sent by sms or e-mail. All patients treated by PTs in private practice receive reminders once a week, up to three times, by sms and e-mail, if they have not answered the questionnaires.

All data are collected electronically using an application run on a tablet or through a web-link. The software is provided by Infopad AS (www.infopad.no). Immediately after the completion of the questionnaires all data are transferred to a secure server with in-memory encryption. All PTs have access to their own patients’ data through Infopad’s web site. Depending on journal system, the PTs can import the patients’ data into their electronic medical journal. Most private practitioners have this possibility. The journal number of the patient is used as the study identifier to enable data flow between the electronic medical journal and the secure Infopad server. The data from all patients are copied from the Infopad server to a secure server for research data at the University of Oslo (Services for Sensitive Data) (Fig. 1). Data management is done according to the quality assurance system of the University of Oslo. We have prepared for linkage between data from FYSIOPRIM and data from national patient registers. This can be done by use of the patient’s journal number to access their unique 11-digit Norwegian personal number. The project is approved by the Regional committee for Medical and Health Research Ethics in Norway (REC no. 2013/2030).

### Variables

An overview of all recorded variables is presented in (Additional file 1: Table S1). For all patients we have included extensive questionnaires to obtain information about demographics, disability and function, pain-related variables, psychosocial factors, treatments and evaluation of treatment as well as response to clinical tests. For adult patients, we also obtain information about employment/work status and psychosocial factors. For subgroups of patients in private practice, we additionally included disease- or region-specific questionnaires. For children below the age of 1 year, we included a detailed infant history and examination, and for school-aged children we included a questionnaire quantifying physical activity. A list of treatment options is completed by the PTs at follow-up (Additional file 2).

### Assessment of representativeness

In order to assess the representativeness of the patients in the FYSIOPRIM database, we compared sex, age and diagnosis/cause of referral in Cohort 1 with aggregated data from HELFO, which registers all patients treated by private practitioners with a reimbursement privilege. For Cohorts 2 and 3 we used aggregated data from Trondheim Municipality for comparisons.

Most adult patients seeking physiotherapy services from private practitioners (Cohort 1) were recruited in Trondheim. We compared sex and age distribution for the 2302 patients registered in FYSIOPRIM Trondheim in the period January 2016 to December 2017 with data from HELFO Trondheim in the period July 2016 to June 2017. Diagnosis by the International Classification of Primary Care (ICPC) codes were available for 1725 of the 2302 patients in Trondheim. Of the 577 patients with missing ICPC code, a large proportion had direct access to a manual therapist and did not have their ICPC code registered.

For adult and older patients receiving physiotherapy services from municipally employed PTs in Trondheim (Cohort 2), we compared data from 68 patients included in FYSIOPRIM with data from Trondheim Municipality for the 72 patients not included in FYSIOPRIM from the end of September to beginning of November 2017. This enabled us to compare sex, age, living conditions, referral entity and cause of referral.

Due to a very low number of children registered in FYSIOPRIM after May 2017, we were not able to use the exact same time period for comparison. Thus, for children receiving physiotherapy services from municipally employed PTs in Trondheim (Cohort 3), data from 162 children included in FYSIOPRIM from May 2016 to April 2017 were compared with data from Trondheim Municipality for all 72 children receiving physiotherapy services from end of September to beginning of November 2017.

### Data preparation and statistical analyses

All the data are collected electronically, thereby avoiding the possibility of mistakes when transferring data from paper to electronic data. Data management is performed using STATA 15 (Stata Corp., College Station, TX, USA). The raw data are stored in a secure server for research data (Services for Sensitive Data). Before data can be used for analyses a range of automated procedures for data preparation and quality checking are performed using standardised written scripts in STATA. Examples are to check i) patterns of missing data, ii) values outside the possible range of a variable (e.g., age > 120 years), iii) conflicting answers (e.g., pregnancy and male), and iv) unexpected frequency distributions for categorical variables. We will explore each variable graphically using histograms, QQ plots and box plots to evaluate the data distribution. Descriptive statistics will be used to describe the patient populations using parametric or non-parametric statistics according to the data distribution. Statistical analyses of associations between variables, prognosis, clinical course and outcome will be detailed in pertinent future publications from the project.

### Organisation of the project

The FYSIOPRIM Research Program has three consortium partners; the University of Oslo, the Norwegian University of Science and Technology and Trondheim Municipality. The FYSIOPRIM steering committee consists of two members from each consortium partner. The committee submits biannual progress reports to the funding body (the Norwegian Fund for Post-Graduate Training in Physiotherapy).

## Representativeness of the database

### Cohort 1: Adult patients seeking physiotherapy services in private practice

Table 1 shows demographics of adult patients seeking physiotherapy services from private practitioners (Cohort 1). Of the 2754 patients registered in the total FYSIOPRIM database by 31.12.2017, 69.2% were females. Age distribution was similar in the age categories up to 70 years, and relatively few patients were above the age of 80 years. The sex and age distributions were fairly similar between the 2302 patients registered in FYSIOPRIM Trondheim and data from HELFO Trondheim encompassing all patients treated by private practitioners with reimbursement privilege in Trondheim in 2016–2017, although patients were somewhat younger in the former (Table 1).

**Table 1:** Demographics of adult patients registered in the FYSIOPRIM database and data from HELFO in Trondheim

	FYSIOPRIM Total 18.06.15–31.12.17 (*n* = 2754)	FYSIOPRIM Trondheim^a^ 01.01.16–31.12.17 (*n* = 2302)	HELFO Trondheim^b^ 01.07.16–30.06.17 (*n* = 19,460)
Variables	n (%)	n (%)	n (%)
Sex			
Female	1851 (69.2)	1605 (69.9)	12,863 (66.1)
Male	825 (30.8)	691 (30.1)	6590 (33.9)
Missing	78	6	7
Age			
18–29 years	466 (16.9)	408 (17.7)	2530 (13.9)
30–39 years	46 (16.9)	382 (16.6)	2385 (13.1)
40–49 years	452 (16.4)	373 (16.2)	2930 (16.0)
50–59 years	482 (17.5)	387 (16.8)	3337 (18.3)
60–69 years	476 (17.3)	385 (16.7)	3316 (18.2)
70–79 years	309 (11.2)	265 (11.5)	2624 (14.4)
80+ years	103 (3.7)	102 (4.4)	1143 (6.3)
Missing	0	0	1195
Specialist^b^			
General PT	1658 (71.7)	1353 (72.7)	12,783 (65.7)
Manual therapist	441 (19.1)	310 (16.6)	5575 (28.7)
Psychomotor PT	215 (9.3)	199 (10.7)	1099 (5.6)
No reimbursement privilege/Missing	440	440	3

*FYSIOPRIM* Research Program for Physiotherapy in Primary Health Care, *HELFO* Norwegian Health Economics Administration, *PT* Physiotherapist

^a^Patients in FYSIOPRIM Trondheim are included in the total FYSIOPRIM database

^b^Data from HELFO Trondheim for all adult patients seeking physiotherapy services from private practitioners with a reimbursement privilege

The distribution of registered diagnostic ICPC code groups in FYSIOPRIM Trondheim was comparable to the data from HELFO Trondheim, apart from a larger proportion of the FYSIOPRIM patients had osteoarthritis, and slightly fewer had neck and low back pain (Table 2).

**Table 2:** Distribution of registered diagnostic ICPC code groups in FYSIOPRIM and HELFO in Trondheim

ICPC category	ICPC diagnosis	ICPC code	FYSIOPRIM Trondheim 01.01.16–31.12.17 (*n* = 1725)	HELFO Trondheim^a^ 01.07.16–30.06.17 (*n* = 19,460)
			n (%)	n (%)
A	General and unspecfied	A01 - A99	53 (3.1)	698 (3.6)
K	Stroke/CVD	K90, K91	13 (0.8)	237 (1.2)
K	Other heart disease	Rest of K-categories	4 (0.2)	173 (0.9)
L	Osteoarthrosis	L89, L90, L91	296 (17.2)	2298 (11.8)
L	Rheumatoid Arthritis	L88	66 (3.8)	464 (2.4)
L	Fract/Sprain/Disloc/Inj	L72 - L81, L96	150 (8.7)	1207 (6.2)
L	Hip pain	L13	59 (3.4)	723 (3.7)
L	Knee pain	L15	62 (3.6)	1093 (5.6)
L	Neck pain	L01, L83	123 (7.1)	2125 (10.9)
L	Low back pain	L02, L03, L84, L85, L86	127 (7.4)	2296 (11.8)
L	Shoulder pain	L08, L92	156 (9.0)	1783 (9.2)
L	Other Musculoskeletal	Rest of L-categories	346 (20.1)	3553 (18.3)
N	Headache	N01, N95	21 (1.2)	254 (1.3)
N	Other Neurology	Rest of N-categories	106 (6.1)	803 (4.1)
P	Psychological	P01 - P99	44 (2.6)	393 (2.0)
R	Respiratory	R01 - R99	38 (2.2)	287 (1.5)
W	Pregnancy, family planning	W01 - W99	30 (1.7)	595 (3.1)
Other	Other ICPC categories	All B, D, F, H, S, T, U, X, Y, Z	31 (1.8)	478 (2.5)

*FYSIOPRIM* Research Program for Physiotherapy in Primary Health Care, *HELFO* Norwegian Health Economics Administration, *ICPC* International Classification of Primary Care

^a^Data from HELFO Trondheim for all adult patients seeking physiotherapy services from private practitioners with a reimbursement privilege

### Cohort 2: Adult and older patients receiving physiotherapy services in Trondheim municipality

Table 3 shows demographics of adult and older patients receiving physiotherapy services from municipally employed PTs in Trondheim (Cohort 2). Of the 655 adult and older patients registered in FYSIOPRIM Trondheim by the end of September 2017, 64.1% were females (Table 3). Most patients (85.6%) were above the age of 70, and 89.7% were living in their own home. In total, 489 (74.7%) received regular physiotherapy, 112 (17.1%) early intervention and 54 (8.2%) ADL rehabilitation/reablement. The most frequent causes of referral to physiotherapy were geriatrics/functional deterioration with or without falls (27.5%), orthopedic (14.7%) and neurologic conditions (11.1%). For this cohort, we compared sex, age, living conditions, referral entity and cause of referral for patients included in FYSIOPRIM in a limited time period with data from Trondheim Municipality for patients not included in FYSIOPRIM during the same time period (last two columns in Table 3). We found that sex and age distribution was similar, but fewer patients not included in FYSIOPRIM Trondheim were living at home and more of them were referred because of geriatrics/functional deterioration (Table 3).

**Table 3:** Demographics of adult and older patients registered in FYSIOPRIM Trondheim and data from Trondheim Municipality

	FYSIOPRIM Trondheim 01.05.16–24.09.17 (*n* = 655)	FYSIOPRIM Trondheim 25.09–03.11.17 (*n* = 68)	Trondheim Municipality^a^ 25.09–03.11.17 (*n* = 72)
Variables	n (%)	n (%)	n (%)
Sex			
Female	419 (64.1)	42 (61.8)	46 (63.9)
Male	235 (35.9)	26 (38.2)	26 (36.1)
Missing	1	0	0
Age			
18–59 years	35 (5.3)	3 (4.4)	6 (8.5)
60–69 years	59 (9.0)	5 (7.4)	9 (12.7)
70–79 years	152 (23.2)	21 (30.9)	14 (19.7)
80–89 years	291 (44.4)	28 (41.2)	34 (47.9)
90+ years	118 (18.0)	11 (16.2)	8 (11.3)
Missing	0	0	1
Living conditions			
In own home	586 (89.7)	63 (92.7)	49 (72.1)
Institution	67 (10.3)	5 (7.4)	19 (27.9)
Missing	2	0	4
Referral entity			
Health and welfare center	150 (22.9)	13 (19.1)	21 (29.2)
Patient/proxy	138 (21.1)	14 (20.6)	18 (25.0)
General practitioner	120 (18.3)	15 (22.1)	6 (8.3)
Home based services	83 (12.7)	12 (17.6)	7 (9.7)
Hospital	82 (12.5)	7 (10.3)	8 (11.1)
Rehabilitation center	28 (4.3)	2 (2.9)	1 (1.4)
Occupational therapist	19 (2.9)	1 (1.5)	4 (5.6)
Multidisciplinary team	13 (2.0)	1 (1.5)	0 (0.0)
Other/unknown	22 (3.4)	3 (4.4)	7 (9.7)
Cause of referral			
Geriatrics/functional deterioration/fall	180 (27.5)	14 (20.6)	25 (34.7)
Orthopedics	96 (14.7)	10 (14.7)	7 (9.7)
Neurology	73 (11.1)	11 (16.2)	9 (12.5)
Musculoskeletal	65 (9.9)	5 (7.4)	5 (6.9)
Heart or lung disease	27 (4.1)	4 (5.9)	2 (2.8)
Cancer	21 (3.2)	4 (5.9)	4 (5.6)
Syndrome	4 (0.6)	0 (0.0)	2 (2.8)
Psychiatry	1 (0.2)	0 (0.0)	2 (2.8)
Early intervention	112 (17.1)	9 (13.2)	5 (6.9)
ADL rehabilitation/reablement	54 (8.2)	8 (11.8)	3 (4.2)
Other/unknown	22 (3.2)	3 (4.4)	8 (11.1)

*FYSIOPRIM* Research Program for Physiotherapy in Primary Health Care

^a^Data from Trondheim Municipality for adult and older patients receiving physiotherapy services from municipally employed physiotherapists and who were not included in FYSIOPRIM

### Cohort 3: Children receiving physiotherapy services in Trondheim municipality

Table 4 shows demographics of children receiving physiotherapy services from municipally employed PTs in Trondheim (Cohort 3). Of the 162 children registered in FYSIOPRIM Trondheim by the end of April 2017, 43.8% were females and 61.1% were below the age of 2 years (Table 4). The most frequent cause of referral to physiotherapy were assessment/guidance related to motor development (32.1%), asymmetrical movement patterns (29.0%) and orthopedic conditions, such as gait and foot alignment (16.0%). To evaluate the representativeness of our child cohort, we compared these proportions with data for all children receiving physiotherapy services in Trondheim Municipality during a limited time period. We found that sex and age distributions as well as causes of referral were highly comparable between the samples (Table 4).

**Table 4:** Demographics of children registered in FYSIOPRIM Trondheim and data from Trondheim Municipality

	FYSIOPRIM Trondheim 01.05.16–30.04.17 (*n* = 162)	Trondheim Municipality^a^ 25.09–03.11.17 (*n* = 72)
Variables	n (%)	n (%)
Sex		
Female	71 (43.8)	28 (38.9)
Male	91 (56.2)	44 (61.1)
Age		
0–1 years	99 (61.1)	41 (56.9)
2–3 years	26 (16.0)	15 (20.8)
4–6 years	12 (7.4)	2 (2.8)
7–9 years	10 (6.2)	7 (9.7)
10–12 years	12 (7.4)	5 (6.9)
13–16 years	3 (1.9)	2 (2.8)
17–18 years	0 (0)	0 (0)
Referral entity		
Primary health care center	82 (50.6)	45 (62.5)
Hospital	29 (17.9)	10 (13.9)
Personnel in kindergarten	24 (14.8)	5 (6.9)
Personnel in school	8 (4.9)	1 (1.4)
School health care services	8 (4.9)	4 (5.6)
Children’s and family’s services	2 (1.2)	1 (1.4)
General practitioner	3 (1.9)	3 (4.2)
Occupational therapist	0 (0)	0 (0)
Proxy/parents	5 (3.1)	3 (4.2)
Other	1 (0.6)	0 (0)
Cause of referral		
Motor development	52 (32.1)	23 (31.9)
Asymmetry (0–1 years)	47 (29.0)	22 (30.1)
Orthopedics (gait, foot alignment)	26 (16.0)	16 (22.2)
Preterm	10 (6.2)	1 (1.4)
Diagnosis/syndrome	5 (3.1)	3 (4.2)
Advice physical activity	5 (3.1)	1 (1.4)
Multidisciplinary assessment	1 (0.6)	1 (1.4)
Heart or lung disease	1 (0.6)	0 (0)
Other	15 (9.3)	5 (6.9)

*FYSIOPRIM* Research Program for Physiotherapy in Primary Health Care

^a^Data from Trondheim Municipality for all children receiving physiotherapy services from municipally employed physiotherapists

## Discussion

In this paper, we describe the design and main features of a longitudinal observational project to build a primary health care physiotherapy database (the FYSIOPRIM database). This large scale prospective physiotherapy project in primary health care in Norway will provide knowledge about the patient groups treated, applied treatments and short- and long-term patient outcome. The design, which involves repeated measurements from baseline and up to 1 year after baseline, enables us to examine the patient’s characteristics, symptoms and disabilities, the patients’ trajectories as well as prognostic factors for favourable outcome.

### Strengths and limitations

The strength of the design is the systematically collected data from baseline throughout the treatment period and beyond, including PROMs, the patients’ and PTs’ main goals and plans for treatment, assessment of goal achievement and outcome. Furthermore, this large database is supported and supplemented by the possibility to link data to local, regional and national registers.

The inclusion of patients is dependent on the cooperation of the PTs. The PTs invite the patients to participate, which is time-consuming in terms of recruitment and data management logistics. Most PTs endure high workloads in their ordinary practice, and implementation of additional electronic patient registrations may be demanding. The effect of this intrusion on their practice will be examined more closely in this project. In order to succeed, we have made extensive efforts into making the system easy, feasible and useful for the clinicians. We have had to balance the amount of variables registered against the time constraints of both PTs and patients. Initially, the project started with an extensive number of variables, but was downscaled in cooperation with the PTs to decrease the burden. One major constraint for the municipally employed PTs is that their journal system is not able to import patient data from the Infopad system. Most PTs in private practice can incorporate data collected through Infopad into their journal system. This enables them to use the information when deciding and evaluating the treatment plan along with their patient. We have also developed summarised reports for individual patient data collected at baseline and end of treatment, providing a quick overview of patient-reported outcomes and changes throughout the treatment period. This information can easily be used in communication with other health care personnel. While it is considered a strength that the patient is involved in the goal-setting process and plans for treatment, some patients may have reported better or different outcomes than they would have done with completely anonymous reporting, which may cause some reporting bias in this project.

There are different sources of possible selection bias in this project. Firstly, participation by the private practitioners is voluntary, and thus they may not be representative of the PTs in their municipalities or in Norway in general. Less than half of the invited PTs chose to participate. The PTs reported different causes as to why they accepted or declined participation. Common explanations for not participating were time-conflicts or technical issues with the electronic registration. Our comparison showed that a larger proportion of patients was treated by psychomotor PTs and a smaller proportion was treated by manual therapists in FYSIOPRIM Trondheim compared with data from HELFO Trondheim (Table 1).

Secondly, the PTs did not include all their patients in the project. Even though more than 100 private practitioners so far have recruited patients, the average number of patients recruited per PT was less than 25 in the 2.5 year period until end of 2017. Municipally employed PTs included less than half of all eligible patients (Table 3). This may be due to time conflicts or that recruitment was simply forgotten in a busy clinical practice. It could also be due to the PT’s personal opinion as to whether a patient was eligible. Even though we have compared our population with external data, we cannot completely exclude that selection biases may have influenced the study population. In the period from June 2015 through December 2017, more than 3000 patients participated in the baseline recording. Our descriptive results at baseline showed that patients seeking physiotherapy services in private practice were largely similar to those in the register from HELFO. The FYSIOPRIM database contains proportionally a larger number of patients with osteoarthritis in private practice, possibly due to the fact that some of the most dedicated PTs performed mostly group-based physiotherapy for this patient group, and thus contributing a large number of patients into the project. A lower proportion of manual therapists in FYSIOPRIM compared with those claiming reimbursement from HELFO may explain the lower rates of neck and low back patients in FYSIOPRIM Trondheim. Also, a larger proportion of the manual therapists did not register their patients’ ICPC code, thus making even fewer patients available for the comparison with HELFO data. Among the patients of municipally employed PTs, sex and age distribution in children, adult and older patients were similar to the aggregated data collected by the municipality in the same time period. A large number of the patients treated by the municipally employed PTs, especially the older patients, were not considered eligible for recruitment for various reasons. As a result, a smaller proportion of patients living in an institution and fewer patients with geriatrics/functional deterioration as the cause of referral were included in FYSIOPRIM.

Thirdly, loss to follow-up and missing data are another concern to the validity of longitudinal time series designs with repeated measurements. This may in particular affect the number of participants with complete trajectories in our project. To evaluate whether differential loss to follow-up occurs, we will compare baseline characteristics of completers with patients lost to follow-up.

### Usability of a primary health care physiotherapy database

This project addresses the lack of comprehensive and systematically collected data for patients receiving physiotherapy services in primary health care in Norway. Several studies are planned, including cross-sectional, longitudinal and methodological studies. The comprehensive baseline assessments enables description of patients’ demographics, general health and quality of life, diagnoses, psychosocial factors, physical function, pain localisation, pain intensity, as well as working ability and sick leave. Furthermore, it enables studies of associations between patient characteristics and treatments and longitudinal assessments of change throughout the treatment course. Moreover, we will be able to identify prognostic factors for improvement for subgroups of patients.

## Conclusion

In this paper we have presented the design and framework of a comprehensive longitudinal observational project in primary care physiotherapy, including characteristics of the participants at baseline. Representativeness was assessed by comparing patients in the FYSIOPRIM database with external populations. These comparisons showed that our patients are largely comparable to the composition of patients treated by both private practitioners and municipally employed PTs in Norway.

## Additional files

Additional file 1: **Table S1**. Overview of variables in the FYSIOPRIM database. (DOCX 127 kb)

Additional file 2: List of treatment options completed by the physiotherapists at follow-up. (DOCX 28 kb)

## Abbreviations

FYSIOPRIM: Research Program for Physiotherapy in Primary Health Care
HELFO: Norwegian Health Economic Administration
PROM: Patient-reported outcome measures
PSFS: Patient-Specific Function Scale
PT: Physiotherapist
