# Supplementary material for: Characteristics, course and outcome of patients receiving physiotherapy in primary health care in Norway: design of a longitudinal observational project
Source: BMC Health Serv Res. 2018 Dec 4;18:936. doi: 10.1186/s12913-018-3729-y (PMC6277993; doi:10.1186/s12913-018-3729-y)
Supplement: Supplementary file 1 — Table S1. Overview of variables in the FYSIOPRIM database. (DOCX 127 kb) [file 12913_2018_3729_MOESM1_ESM.docx]

**Additional File 1.**

**Table A1.** Overview of variables in the FYSIOPRIM database.

| **Name of variable** | **Content description or wording of variable** | **Response option and scoring** | | | **Data type** | **Time points** | | | | | **Patients** | | |
| --- | --- | --- | --- | --- | --- | --- | --- | --- | --- | --- | --- | --- | --- |
|  |  |  |  |  | **S=String**  **Ca=Categorical**  **Co=Continuous** | **Baseline** | **1 m** | **3m** | **6m** | **12m** | **Cohort 1** | **Cohort 2** | **Cohort 3** |
| **Demographics** | | | | | | | | | | | | | |
| Sex | Sex of patient | Female  Male | | | Co | x |  |  |  |  | x | x | x |
| Age | Age of patient | Age in years | | | Ca | x |  |  |  |  | x | x | x |
| Referral | Is the patient referred? | Yes  No | | | Ca | x |  |  |  |  | x |  |  |
| ICPC code | ICPC code from referral entity | ICPC category (letter) and code (number) | | | Ca | x |  |  |  |  | x |  |  |
|  | ICPC code from the therapist | Free text | | | S |  | x | x |  |  | x |  |  |
| Referral entity | From where is the patient referred? | List of different referral entities (for example general practitioners and specialists) | | | Ca | x |  |  |  |  | x | x | x |
| Cause of referral | What is the cause of referral? | List of different causes (for example neurology and musculoskeletal disorders) | | | Ca | x |  |  |  |  |  | x | x |
| Date of referral | Date of received referral | Date (dd/mm/yyyy) | | | Co | x |  |  |  |  |  | x | x |
| Date of first contact | Date of first contact with the physiotherapist | Date (dd/mm/yyyy) | | | Co | x |  |  |  |  |  | x | x |
| Waiting time | How long has it been since the patient contacted a physiotherapist for an appointment? | <1 week  1-2 weeks  2-4 weeks  4 weeks-3 months | 3-6 months  6-12 months  >1 year | | Ca | x |  |  |  |  | x |  |  |
| Priority-assignment | The physiotherapist’s priority-assignment (1-4) | 1. Quick start of intervention is essential and waiting time will lead to negative consequences  2. Good treatment outcome is associated with early intervention  3. Intervention is expected to have good effect, but start of treatment can wait up to 1 month  4. Intervention is expedient for maintenance and improvement of activities of daily living, but start of intervention can wait without any significant consequences | | | Ca | x |  |  |  |  | x | x | x |
| Body regions | Mark the relevant body region(s) for your complaint (multiple answers possible) | Head  Jaw  Columna  Neck  Thorax  Low back  Pelvis | Tailbone  Shoulder/upper arm  Hand/forearm  Hip  Knee  Ankle/foot  Widespread/complex pain | | Ca | x |  |  |  |  | x | x | x |
| Comorbidity | Specify diagnosis (if applicable)  (multiple answers possible) | Myalgia  Tendinopathy  Pain > 6 months  Mental health Arthrosis Rheumatology  Heart disease Asthma/lung disease  Stroke  Neurology (central or peripheral)  Cancer | Lymphedema Headache/migraine  Dizziness  Trauma ligament  Trauma fracture  Trauma muscles  Surgery/post-surgery rehabilitation  Elderly with reduced function  Dementia/cognitive deficit | | Ca | x |  |  |  |  | x | x | x |
| Body height | Height in centimeters (cm) | Cm | | | Co | x |  |  |  |  | x | x | x |
| Body weight | Weight in kilograms (kg) | Kg | | | Co | x |  |  |  |  | x | x | x |
| Smoking | Do you smoke? | Yes  No | | | Ca | x |  |  |  |  | x | x |  |
| Education | Your highest level of education | Primary school or lower  High school  Up to 4 years of college/university  More than 4 years of college/university | | | Ca | x |  |  |  |  | x | x |  |
| Ethnicity | Where are you born? | Norway  Europe  Africa  Asia | North America  South or middle America  Oceania | | Ca | x |  |  |  |  | x | x | x |
| Marital status | Marital status | Married/cohabitant  Divorced  Widow/widower  Single | | | Ca | x |  |  |  |  | x | x |  |
| Living situation | Living situation | Own home alone  Own home with others  Institution | | | Ca | x |  |  |  |  | x | x |  |
| Care of children | How many children below 18 years do you have daily care for? | Number of children | | | Ca | x |  |  |  |  | x | x |  |
| Family and friends | 1. Do you have family or friends who can offer you help when needed?  2. Do you have family and friends with whom you can talk confidentially? | Yes  No  Yes  No | | | Ca | x |  |  |  |  |  | x |  |
| Health services | Have you received treatment for your complaints during the last 12 months? | No  General practitioner  Physician, specialist  Chiropractor  Manual therapist  Physiotherapist | Psychomotor physiotherapist  Psychologist  Alternative/  complementary therapist | | Ca | x |  |  |  |  | x | x | x |
| Main treatment goal | Main treatment goal  (set by patient/therapist collaboration, evaluated at follow-up) | Free text | | | S | x | x | x | x |  | x | x | x |
| Main treatment | Main treatment (set by patient/therapist collaboration, compliance evaluated at follow-up) | Free text | | | S | x | x | x | x |  | x | x | x |
| **Disability and function** | | | | | | | | | | | | | |
| Physiotherapist’s diagnosis | Functional diagnosis defined by the physiotherapist | Free text | | | S | x |  |  |  |  | x | x | x |
| PSFS^1^ | Patient-Specific Functional Scale; 3 activities defined by the patient in collaboration with the physiotherapist | NRS: 0-10; 0=not able to perform activity and 10=no problem to perform activity | | | Co | x | x | x | x | x | x | x |  |
| EQ5D-5L^2^ | EuroQol - Health related quality of life; 5 items of mobility, self- care, usual activities, pain and anxiety/depression | Each item have 5 response categories  Summarized as index score: -0.59-1.00; 0=death and 1=full health (negative scores=worse than death) | | | Ca  Co | x |  | x | x | x | x | x |  |
| 15D^3^ | Health related quality of life; 15 items of mobility, vision, hearing, breathing, sleeping, eating, speech, excretion, usual activities, mental function, discomfort and symptoms, depression, vitality, sexual activity | Each item have 5 response categories  Summarized as index score: 0-1; 0=death and 1=full health | | | Ca  Co | x |  | x | x | x | x |  |  |
| COOP/  WONCA^4^ | 6 items of physical fitness, feelings, daily activities, social activities, change in health and overall health | 5-6 response categories varying according to item | | | Co | x |  |  |  |  | x |  |  |
| ADL | Due to pain or complaints, how much reduced is your activities of daily life? | Very much reduced  Quite reduced  Slightly reduced  Not reduced | | | Ca | x |  |  |  |  | x | x | x |
| Walking aid | Do you use any walking aids? | Yes  No | | | Ca | x |  |  |  |  | x | x | x |
| Time spent sitting | How many hours during a regular day do you spend sitting? (Both work and leisure time) | Range: 0-24 hours | | | Co | x |  |  |  |  | x |  |  |
| Physical activity frequency^5^ | How often do you exercise during a week? | Never  Once  Less than once | 2-3 times  Approximately each day | | Ca | x |  | x | x | x | x | x |  |
| Physical activity intensity^5^ | How hard do you exercise? | Easy without sweating and getting breathless  Getting breathless and sweating  Almost completely to exhaustion | | | Ca | x |  | x | x | x | x | x |  |
| Physical activity duration^5^ | For how long do you exercise each time? | Less than 15 min  15-29 min | 30 min-1 h  >1 h | | Ca | x |  | x | x | x | x | x |  |
| Physical activity for children >7 years | 12 questions of daily activity including frequency and duration | Frequency: Daily, 4-6x/week, 2-3x/week, 1x/week, 1x/month, never | Duration: None, 1 hour, 2-3 hours, 4-6 hours, >7 hours | | Ca | x |  |  |  |  |  |  | x |
| Infant’s anamnesis for children <1 year | Birth history, regulatory difficulties, asymmetry in postures or movements | Different response categories according to domains, mainly identification of risk factors, and differentiation of normal versus abnormal findings | | | Ca | x |  |  |  |  |  |  | x |
| Risk of falling | Ten questions regarding risk factors for fall:  1. Previous falls last year?  2. Problems with balance or walking?  3. Unable to raise from sitting to standing without use of hands?  4. Parkinson disease or stroke?  5. Three or more chronic diseases?  6. >5 medications per day?  7. Reduced cognitive function?  8. Reduced vision?  9. Body mass index <20?  10. Fear or worry of falling? | Question 1-9:  Yes  No | Question 10:  Not at all worried  Slightly worried  Quite worried  Very worried | | Ca | x |  |  |  |  |  | x |  |
| Floor to standing mobility | 1. Can you get down on the floor on your own?  2. Can you get up from the floor on your own? | Yes  No  Do not know | | | Ca | x |  |  |  |  |  | x |  |
| **Employment/work** | | | | | | | | | | | | | |
| Employment status | What is your current employment situation? (multiple answers possible) | Paid work  Student  Retired  Disability pension | Work allowance pension  Non-paid work  Unemployed | | Ca | x |  | x | x | x | **x** | **x** |  |
| Employment percentage | Specify the percentage of employment | Range: 0-100 % |  | | Co | x |  | x | x | x | x | x |  |
| Sick leave percentage | Specify the percentage of sick leave | Range: 0-100 % |  | | Co | x |  | x | x | x | x | x |  |
| Disability pension percentage | Specify the percentage of disability pension | Range: 0-100% |  | | Co | x |  | x | x | x | x | x |  |
| Work ability index-1 item^6^ | What is your current work ability compared with the lifetime best? | NRS: 0-10; 0=cannot work and 10=working at best | | | Co | x |  | x | x | x | x | x |  |
| **Pain-related variables** | | | | | | | | | | | | | |
| Current pain intensity | Mark the number that indicate your current pain intensity | NRS: 0-10; 0=no pain and 10=worst imaginable pain | | | Co | x | x | x | x | x | x |  | x |
| Pain intensity last week^7^ | Item 2 from Örebro Screening Questionnaire: How would you rate the pain that you have had during the past week? | NRS: 0-10; 0=no pain and 10=worst imaginable pain | | | Co | x |  | x | x | x | x | x |  |
| Pain duration^7^ | Item 1 from Örebro Screening Questionnaire: How long have you had your current pain problem? | 0-1 week  1-2 weeks  3-4 weeks  4-5 weeks  6-8 weeks | 9-11 weeks  3-6 months  6-9 months   - 1. months   >1 year | | Ca | x |  |  |  |  | x | x |  |
| Pain drawing | “Mark areas in the body drawing where you have pain” | Number of marked areas, range: 0-112 | | | Co | x |  | x | x | x | x | x |  |
| Temporal aspects of pain | 1. Is the pain continuous?  2. Does the pain intensity vary?  If yes on 2:  3. Describe the temporal aspects of the pain: | Yes, I can feel it all the time  No, it is on and off  Yes  No, it is stable  It varies during the day and night  It varies from day to day  Other | | | Ca | x |  | x | x | x | x | x |  |
| Start of complaint | How did your complaints start? | Acute  Gradually | | | Ca | x |  |  |  |  | x |  |  |
| Analgesics | Have you used pain medication the last week?  If yes:  What medicines? (Three categories defined from active ingredient: Based on paracetamol, acetylsalisylacid or ibuprofen) | Yes  No  1. Paracetamol, Paracet, Panodil, Pimol, Pinex or Perfalgan  2. Albyl-E (500 mg), Aspirin, Bloboid or Dispril  3. Ibuprofen, Ibux, Ibuprox, Ibumetin, Brufen, Naproxen, Naprosyn  4. Others | | | Ca | x |  | x | x | x | x | x |  |
| Medicines | Number of daily medicines | 0  1-4  5 or more | | | Ca | x |  | x | x | x | x | x |  |
| **Psychosocial factors** | | | | | | | | | | | | | |
| HSCL-10^8^ | Hopkins Symptom Check List; 10 item version | Range: 1-4; 1=not at all and 4=very much (extremely), mean item score is calculated | | | Co | x |  | x | x | x | x |  |  |
| Fear of movement^9^ | Single item: How much fear do you have that these complaints would be increased by physical activity? | NRS: 0-10; 0=no fear and 10=very much fear | | | Co | x |  |  |  |  | x | x |  |
| TSK^10^ | Tampa Scale of Kinesiophobia; 13 item version | Range: 13-53; higher score indicates higher level of kinesiophobia | | | Co | x |  |  |  |  | x |  |  |
| Örebro-10 item^7^ | Örebro Screening Questionnaire; 10 item version | Range: 0-100; higher score indicates higher levels of estimated risk for developing pain related disability | | | Co | x |  |  |  |  | x |  |  |
| Pain self-efficacy^11^ | Pain Self Efficacy Questionnaire; 2 items:  1. I can do some form of work, despite pain (work includes housework and paid and unpaid work)  2. I can live a normal lifestyle, despite pain | Range: 0-6; 0=not at all confident and 6=completely confident, total range: 0-12; higher score indicates higher level of self-efficacy | | | Co | x |  | x | x | x | x |  |  |
| Treatment expectancy^12^ | I believe physiotherapy will improve my function | Totally agree  Agree  Neither agree nor disagree  Disagree  Totally disagree | | | Ca | x |  |  |  |  | x | x | x |
| BACQ^13^ | The Brief Approach/Avoidance Coping Questionnaire | Range: 1-5; 1=disagree completely and 5=agree completely, higher score indicates use of more approach-oriented coping strategies | | | Co | x |  | x | x | x | x |  |  |
| Family and friends | 1. Do you have family or friends that can give you help when needed?  2. Do you have family and friends with whom you can talk confidentially? | Yes  No  Yes  No | | | Ca | x |  |  |  |  |  | x |  |
| Short FES-I^14^ | The Short Falls Efficacy Scale-Internationally | Range: 7-28; higher score indicates severe concern about falling | | | Co | x |  |  |  |  |  | x |  |
| **Disease- or region-specific questionnaires** | | | | | | | | | | | | | |
| NDI^15,16^ | Neck Disability Index | Range: 0-100; 0=no disability and 100=100% disability | | | Co | x |  | x | x | x | x |  |  |
| ODI^17^ | Oswestry Disability Index | Range: 0-100; 0=no disability and 100=100% disability | | | Co | x |  | x | x | x | x |  |  |
| STarTBack^18^ | Keele STarTBack screening tool, subgrouping of patients into 3 a priori treatment options | Range: 0-9 points, categorisation into risk groups based on the scoring | | | Ca | x |  |  |  |  | x |  |  |
| Hannover^19^ | Hannover Functional Ability Questionnaire; 12 items | Range: 0-24; higher score indicates poorer function | | | Co | x |  | x | x | x | x |  |  |
| SPADI^20^ | Shoulder Pain and Disability Index | Range: 0-100; 0=no disability and 100=100% disability | | | Co | x |  | x | x | x | x |  |  |
| Quick-DASH^21^ | Shortened version of the Disabilities of the Arm, Shoulder and Hand Score; 11 items | Range: 0-100; 0=no disability and 100=100% disability | | | Co | x |  | x | x | x | x |  |  |
| PGQ^22^ | Pelvic Girdle Questionnaire; 25 items | Range: 0-100; 0=no problem and 100=to a large extent | | | Co | x |  | x | x | x | x |  |  |
| HOOS^23^ | The Hip Disability and Osteoarthritis Outcome Score | Subscores for five domains:  Pain: 0-100  Symptoms: 0-100  ADL: 0-100  Sports/Recreation: 0-100  QOL: 0-100 | | | Co | x |  | x | x | x | x |  |  |
| KOOS^24^ | The Knee injury and Osteoarthritis Outcome Score | Subscores for five domains:  Pain: 0-100  Symptoms: 0-100  ADL: 0-100  Sports/Recreation: 0-100  QOL: 0-100 | | | Co | x |  | x | x | x | x |  |  |
| OA-QI^25^ | Arthrosis Quality Indicator;  17 questions about treatment alternatives for osteoarthritis | 3 response categories; yes, no and a third that varies between the questions, pass rates for each question are calculated and used as a quality indicator | | | Co | x |  |  |  |  | x |  |  |
| PCS^26^ | Pain Catastrophizing Scale | Range: 0-52; higher score indicates higher level of catastrophising | | | Co | x |  | x | x | x | x |  |  |
| Physical functioning from SF-36^27^ | 10 items of physical functioning  from SF-36 related to activities in daily life | Range: 1-3 for each item; 1=yes, limited a lot and 3=no, not limited at all, domain score range: 0-100%; higher score indicates better function | | | Co | x |  | x | x | x | x |  |  |
| Vitality from SF-36^27^ | 4 items of vitality from SF-36 related to activities in daily life | Range: 1-6 for each item; 1=all the time and 6=not at all, domain score range: 0-100%; higher score indicates better function | | | Co | x |  | x | x | x | x |  |  |
| ISI^28^ | Insomnia Severity Index; 7 items | Range: 0-4 for each item; 0=not at all and 4=extremely, total range: 0-28; higher score indicates greater insomnia severity | | | Co | x |  | x | x | x | x |  |  |
| Norwegian questionnaire for the assessment of body experience^29^ | 64 items; 11 subscales of body awareness, body contact, body endurance, body satisfaction, balance, breathing, aversion, tension, bodily discomfort, distance/remoteness and physical distance/boundaries | Range: 1-6 for each item; 1=never and 6=all the time (scores for some items are reversed), subscale scores are mean of item scores within the subscale | | | Co | x |  |  |  |  | x |  |  |
| **Clinical tests** | | | | | | | | | | | | | |
| Back Performance Scale^30^ | Performance of five different tests: Sock-, pick-up-, roll-up-, fingertip to floor and lift test | Range: 0-3 for each test, total range: 0-15; higher score indicates poorer performance | | | Co | x |  | x |  |  | x |  |  |
| Tests of physical fitness^31^ | 6 min walk test  Stairs  Grip strength  30 s sit to stand | Time (sec)  Time (sec)  Kilograms (kg)  Repetitions (n) | | | Co | x |  | x |  |  | x |  |  |
| P4 test^32^ | Posterior Pelvic Pain Provocation test | No pain  Pain (relevant)  Pain (irrelevant) | | | Ca | x |  | x |  |  | x |  |  |
| ASLR^33^ | Active Straight Leg Raise | Range: 0-5; 0=no problem and 5=impossible to raise leg | | | Ca | x |  | x |  |  | x |  |  |
| TUG^34^ | Timed Up and Go | Time (sec) | | | Co | x |  | x | x |  | x | x |  |
| Shoulder tests | Hawkins test  Active range of motion  Isometric tests | Positive  Negative | | | Ca | x |  | x |  |  | x |  |  |
| SPPB^35^ | Short Physical Performance Battery; 4 tests:  Tandem standing 10 sec  Semi-tandem standing 10 sec  Gait speed 4 m  Sitting to standing | Performance on the tests are categorised according to time used (see SPPB manual for information)  Seconds  Seconds  Seconds  Seconds | | | Ca | x |  | x |  |  | x | x |  |
| BBS^36^ | The Berg Balance Scale; 14 items | Range: 0-56; higher score indicates lower risk of fall | | | Co | x |  |  | x |  |  | x |  |
| Clinical examination of infants (<1 year) | Observation, postural reactions, palpation, neurological and orthopedic tests, active and passive movements | Different response categories depending on area examined, mainly differentiation of normal versus abnormal findings | | | Ca | x |  |  |  |  |  |  | x |
| **Treatments** | | | | | | | | | | | | | |
| Treatments | List of the main physiotherapy treatment options | See Additional File 2 for details | | | Ca |  | x | x | x |  | x | x | x |
| Collaboration | Collaboration and interaction with other health care services and personnel | Collaborator: General practitioner, specialist, others  Type of collaboration: Report, phone call, digital message, other  Referral: Specialist, other physiotherapist, sick leave and imaging/other examinations (manual therapists only), other | | | Ca |  | x | x | x |  | x | x | x |
| **Evaluation of treatment** | | | | | | | | | | | | | |
| GPE^37^ | Global Perceived Effect (1-7) | 1=very much better  2=much better  3=slightly better  4=neither better nor worse | | 5=slightly worse  6=much worse  7=very much worse | Ca |  |  | x | x | x | x | x | x |
| Fulfillment of treatment expectations | To what degree where your expectations to physiotherapy fulfilled? | To a very large degree  To a large degree  To some degree  To a little degree  To a very little degree | | | Ca |  |  | x | x | x | x | x | x |
| Benefit of treatment | To what degree have you benefited from physiotherapy? |  |  |  |  |  |  | x | x | x | x | x | x |

Cohort 1= Adult patients seeking physiotherapy services from private practitioners

Cohort 2= Adult and older patients receiving physiotherapy services from municipally employed physiotherapists

Cohort 3= Children receiving physiotherapy services from municipally employed physiotherapists

FYSIOPRIM = Research Program for Physiotherapy in Primary Health Care

ICPC = International Classification of Primary Care

NRS = Numeric Rating Scale

**References**

1. Stratford P, Gill C, Westaway M, Binkley J. Assessing Disability and Change on Individual Patients: A Report of a Patient Specific Measure. Physiotherapy Canada 1995;47:258-63.
2. Solberg TK, Olsen JA, Ingebrigtsen T, Hofoss D, Nygaard OP. Health-related quality of life assessment by the EuroQol-5D can provide cost-utility data in the field of low-back surgery. Eur Spine J 2005;14:1000-7.
3. Sintonen H. The 15D instrument of health-related quality of life: properties and applications. Annals of medicine 2001;33:328-36.
4. Bentsen BG, Natvig B, Winnem M. [Assessment of one's own functional status. COOP-WONCA questionnaire charts in clinical practice and research]. TidsskrNor Laegeforen 1997;117:1790-3.
5. Kurtze N, Rangul V, Hustvedt BE, Flanders WD. Reliability and validity of self-reported physical activity in the Nord-Trondelag Health Study: HUNT 1. Scandinavian journal of public health 2008;36:52-61.
6. de Zwart BC, Frings-Dresen MH, van Duivenbooden JC. Test-retest reliability of the Work Ability Index questionnaire. OccupMed(Lond) 2002;52:177-81.
7. Linton SJ, Nicholas M, MacDonald S. Development of a short form of the Orebro Musculoskeletal Pain Screening Questionnaire. Spine (Phila Pa 1976) 2011;36:1891-5.
8. Derogatis LR, Lipman RS, Rickels K, Uhlenhuth EH, Covi L. The Hopkins Symptom Checklist (HSCL). A measure of primary symptom dimensions. Modern problems of pharmacopsychiatry 1974;7:79-110.
9. Verwoerd AJ, Luijsterburg PA, Timman R, Koes BW, Verhagen AP. A single question was as predictive of outcome as the Tampa Scale for Kinesiophobia in people with sciatica: an observational study. J Physiother 2012;58:249-54.
10. Haugen AJ, Grovle L, Keller A, Grotle M. Cross-cultural adaptation and validation of the Norwegian version of the Tampa scale for kinesiophobia. Spine (Phila Pa 1976) 2008;33:E595-601.
11. Nicholas MK, McGuire BE, Asghari A. A 2-item short form of the Pain Self-efficacy Questionnaire: development and psychometric evaluation of PSEQ-2. The journal of pain : official journal of the American Pain Society 2015;16:153-63.
12. Bishop MD, Bialosky JE, Cleland JA. Patient expectations of benefit from common interventions for low back pain and effects on outcome: secondary analysis of a clinical trial of manual therapy interventions. J Man Manip Ther 2011;19:20-5.
13. Finset A, Steine S, Haugli L, Steen E, Lærum E. Approach/Avoidance Coping Questionnaire: development and validation. Psychology, Health & Medicine 2002;7:75-85.
14. Kempen GI, Yardley L, van Haastregt JC, et al. The Short FES-I: a shortened version of the falls efficacy scale-international to assess fear of falling. Age Ageing 2008;37:45-50.
15. Kovacs FM, Abraira V, Royuela A, et al. Minimum detectable and minimal clinically important changes for pain in patients with nonspecific neck pain. BMC Musculoskelet Disord 2008;9:43.
16. Vernon H. The Neck Disability Index: state-of-the-art, 1991-2008. J Manipulative Physiol Ther 2008;31:491-502.
17. Grotle M, Brox JI, Vollestad NK. Cross-cultural adaptation of the Norwegian versions of the Roland-Morris Disability Questionnaire and the Oswestry Disability Index. J Rehabil Med 2003;35:241-7.
18. Hill JC, Dunn KM, Lewis M, et al. A primary care back pain screening tool: identifying patient subgroups for initial treatment. Arthritis Rheum 2008;59:632-41.
19. Magnussen L, Strand LI, Lygren H. Reliability and validity of the back performance scale: observing activity limitation in patients with back pain. Spine (Phila Pa 1976) 2004;29:903-7.
20. Roach KE, Budiman-Mak E, Songsiridej N, Lertratanakul Y. Development of a shoulder pain and disability index. Arthritis Care Res 1991;4:143-9.
21. Beaton DE, Wright JG, Katz JN. Development of the QuickDASH: comparison of three item-reduction approaches. J Bone Joint Surg Am 2005;87:1038-46.
22. Stuge B, Garratt A, Krogstad JH, Grotle M. The pelvic girdle questionnaire: a condition-specific instrument for assessing activity limitations and symptoms in people with pelvic girdle pain. Physical Therapy 2011;91:1096-108.
23. Nilsdotter AK, Lohmander LS, Klassbo M, Roos EM. Hip disability and osteoarthritis outcome score (HOOS)--validity and responsiveness in total hip replacement. BMC musculoskeletal disorders 2003;4:10.
24. Roos EM, Lohmander LS. The Knee injury and Osteoarthritis Outcome Score (KOOS): from joint injury to osteoarthritis. Health and quality of life outcomes 2003;1:64.
25. Østerås N, Garratt A, Grotle M, et al. Patient-reported quality of care for osteoarthritis: development and testing of the osteoarthritis quality indicator questionnaire. Arthritis care & research 2013;65:1043-51.
26. Fernandes L, Storheim K, Lochting I, Grotle M. Cross-cultural adaptation and validation of the Norwegian pain catastrophizing scale in patients with low back pain. BMC Musculoskelet Disord 2012;13:111.
27. Ware JE, Jr., Sherbourne CD. The MOS 36-item short-form health survey (SF-36). I. Conceptual framework and item selection. Medical care 1992;30:473-83.
28. Bastien CH, Vallieres A, Morin CM. Validation of the Insomnia Severity Index as an outcome measure for insomnia research. Sleep medicine 2001;2:297-307.
29. Pedersen G, Tybring-Pedersen K, Tønder M, Arnestad TB, Møller AC, Øya T. Spørreskjema for måling av kroppsopplevelse. Tidsskrift for Norsk Psykologforening 2013;50:992-8.
30. Strand LI, Moe-Nilssen R, Ljunggren AE. Back Performance Scale for the assessment of mobility-related activities in people with back pain. PhysTher 2002;82:1213-23.
31. Tveter AT, Dagfinrud H, Moseng T, Holm I. Measuring health-related physical fitness in physiotherapy practice: reliability, validity, and feasibility of clinical field tests and a patient-reported measure. J Orthop Sports Phys Ther 2014;44:206-16.
32. Östgaard HC, Zetherstrom G, Roos-Hansson E. The posterior pelvic pain provocation test in pregnant women. European Spine Journal 1994;3:258-60.
33. Mens JM, Vleeming A, Snijders CJ, Stam HJ, Ginai AZ. The active straight leg raising test and mobility of the pelvic joints. European Spine Journal 1999;8:468-73.
34. Podsiadlo D, Richardson S. The timed "Up & Go": a test of basic functional mobility for frail elderly persons. J Am Geriatr Soc 1991;39:142-8.
35. Guralnik JM, Simonsick EM, Ferrucci L, et al. A short physical performance battery assessing lower extremity function: association with self-reported disability and prediction of mortality and nursing home admission. J Gerontol 1994;49:M85-94.
36. Berg KO, Wood-Dauphinee SL, Williams JI, Maki B. Measuring balance in the elderly: validation of an instrument. Can J Public Health 1992;83 Suppl 2:S7-11.
37. Dworkin RH, Turk DC, Farrar JT, et al. Core outcome measures for chronic pain clinical trials: IMMPACT recommendations. Pain 2005;113:9-19.
