# Supplementary material for: Characteristics, course and outcome of patients receiving physiotherapy in primary health care in Norway: design of a longitudinal observational project
Source: BMC Health Serv Res. 2018 Dec 4;18:936. doi: 10.1186/s12913-018-3729-y (PMC6277993; doi:10.1186/s12913-018-3729-y)
Supplement: Supplementary file 2 — List of treatment options completed by the physiotherapists at follow-up. (DOCX 28 kb) [file 12913_2018_3729_MOESM2_ESM.docx]

**Additional File 2.** List of treatment options completed by the physiotherapists at follow-up.

Check the different treatment and treatment modalities performed from start of the treatment to the current evaluation.

**New examination/re-evaluation**

Yes

No

**Advice and guidance (multiple answers possible)**

Rest and/or relief

Physical activity

Importance of daily activity

Avoid unfavorable movements or activities

Normal activity, but take pain into account

Normal activity despite pain

Reduce fear of movement

Ergonomics or work adaptation

Posture

Diet, weight regulation

Sleep

Sick leave

Information about organisations, web sites, activities other places

Other, specify:

**ADL (Activity of daily living) (multiple answers possible)**

Displacement training

Walking

Walking stairs

Activities of daily life

Other, specify:

**Adaptations/aids (multiple answers possible)**

Facilitation and help with activities or aids

Ordering, testing and training of aids

Work place evaluation

**Exercises**

Check the exercises performed by the patient (including guided exercises, self-training or home exercises). Check the most frequently used exercise intensity and report the mean number of days per week for strength and cardiorespiratory exercises.

*Definition of intensity*

Light: Borgs scale 6-11 (very, very easy to easy)

Moderate: Borgs scale 12-15 (somewhat hard to hard)

Hard: Borgs scale 16-20 (very hard to maximal exertion)

|  | Light | Moderate | Hard | Days per week (1-7) |
| --- | --- | --- | --- | --- |
| Functional strength |  |  |  |  |
| Isometric strength |  |  |  |  |
| Dynamic strength |  |  |  |  |
| Cardiorespiratory |  |  |  |  |

**Other exercises (multiple answers possible)**

Balance\coordination\stability

Mobilisation exercises

Stretching exercises

Relaxation\tension reducing exercises

Consciousness of posture

Mindfulness

Other, specify:

**Pain**

Check whether the patient has had pain that has influenced the exercises and with what degree of pain the patient has performed the exercises

**The patient has pain that influences the exercises**

Yes

No

If yes:

**The exercises are performed with the following degree of pain**

NRS 0-10, where 0 = no pain and 10 = worst imaginable pain

0 (no pain)

1-3 (low pain)

4-5 (moderate pain)

6-10 (high pain)

**Manual techniques (multiple answers possible)**

Joint mobilisation

Manipulation

Massage or soft tissue treatment

Treatment grips

Positioning

Stretching

Trigger point treatment

Dry needling

Other, specify:

**Apparatus (multiple answers possible)**

Ultrasound

Electrotherapy

Shock wave therapy

Other, specify:

**Psychomotor physiotherapy**

Check this box if the patient has received psychomotor physiotherapy and specify content

**Most of the treatment has been:**

Individual

Group-based

Self-training
